# Friends, relatives, sanity, and health: The costs of politics

**DOI:** 10.1371/journal.pone.0221870

**Published:** 2019-09-25

**Authors:** Kevin B. Smith, Matthew V. Hibbing, John R. Hibbing

**Affiliations:** 1 Department of Political Science, University of Nebraska-Lincoln, Lincoln, NE, United States of America; 2 Department of Political Science, University of California-Merced, Merced, CA, United States of America; Augusta University, UNITED STATES

## Abstract

Political scientists have long known that political involvement exacts costs but they have typically defined these costs in relatively narrow, largely economic terms. Though anecdotal evidence suggests that the costs of politics may in fact extend beyond economics to frayed personal relationships, compromised emotional stability, and even physical problems, no systematic evidence on these broader costs exists. We construct and validate batteries of survey items that delineate the physical, social, and emotional costs of political engagement and administer these items to a demographically representative sample of U.S. adults. The results suggest that a large number of Americans believe their physical health has been harmed by their exposure to politics and even more report that politics has resulted in emotional costs and lost friendships.

## Introduction

Like all choices, the decision of whether or not to get involved in politics can be viewed in cost-benefit terms. Downs’ [1] classic formulation of political participation began with a somewhat narrow conceptualization. Costs were taken to be the time, effort, and money required to become informed and then to participate in politics, perhaps by traveling to the polls, making a campaign contribution, or communicating with a public official; benefits were the favorable policies enacted as a result of a preferred candidate assuming office.

In order to explain a variety of political phenomena, the benefits of politics were quickly expanded to include social and psychological as well as policy benefits. These additional benefits included feelings of fulfillment derived from performing a civic duty [2–3], expressive or solidary benefits produced by joining with like-minded people to support a candidate, party, or cause [4–5], and the entertainment rewards derived from being a “hobbyist” [6]. In addition, research has provided evidence that political participation can positively affect social capital, a collective benefit that not only promotes social cohesion but may even have public health benefits (e.g. [7–8]). Though the benefits of political engagement have broadened and expanded, the costs continue to be viewed in narrow, functional, tangible, and often economic terms. This is unfortunate because, particularly in light of the emotionally-wrenching, increasingly polarized climate of the last few decades, it has become clear that the costs of politics entail more than carving out time to acquire political information or paying a babysitter on Election Day. People often pay social, psychological, emotional, and even physical costs as a result of political engagement, but the extent of these costs and their specific nature and implications have, to the best of our knowledge, never been systematically measured or investigated in large part because suitable instruments have never been developed.

That such costs exist is not in question. Damaged friendships, ruined family reunions, and disrupted workplaces, not to mention feelings of guilt, regret, frustration, anguish, and remorse, have all been attributed to political differences. These sorts of psychological stressors are suspected to underlie a range of health problems believed to accompany divisive electoral campaigns, especially when vulnerable populations perceive themselves as targeted [9–10]. There is little doubt that politics is a prominent cause of stress for modern American adults. A recent survey by the American Psychological Association [11] found that 57 percent of Americans identified politics as a very or somewhat significant source of stress, and other polls report that the most commonly expressed fears of Americans center on government and politics [12–13]. The few clinical studies on the health impacts of politics that do exist suggest such effects may be all too real. The polarizing presidential election of 2016 appears to have triggered clinical levels of distress in at least one college population [14], and may even be linked to death rate fluctuations in some geographic areas [15]. More anecdotally, in the wake of the 2016 election, clinical psychologists reported a jump in mental health pathologies directly attributed to politics—what the media have termed “election stress disorder” [16].

In short, there is clearly an empirical basis for the hypothesis that politics, especially the divisive sort of politics characterizing the contemporary civic sphere in the United States, negatively affects social, psychological, and even physical health. Yet there is remarkably little systematic data on the existence and extent of these costs. How widespread are they? Are the costs primarily social and emotional? Do they extend to the physical realm? What kind of person is most likely to believe they suffer as a result of their political involvement? Despite indications that such costs may be real and broadly felt, there is no systematic empirical basis for answering any of these questions.

Our specific purpose in this paper is to begin addressing these questions by 1.) constructing and validating survey instruments suitable for assessing the specific costs of politics, thereby eventually making it possible to measure variations over time and across political cultures, 2.) providing the first systematic assessment of the impact of political involvement on social, psychological and physical health at the time our survey was administered, and 3.) identifying the type of person most likely to suffer psychological, emotional, social, and physical costs as a result of politics, again at one point in time.

## Materials and methods

To achieve these goals we administered a series of specially drafted survey items to a representative sample of American adults. Data were collected by YouGov from March 15 to March 20, 2017. YouGov uses an online panel of approximately 1.8 million US respondents to create representative samples. Our sample was specifically matched to a 2010 American Community Survey (ACS) sampling frame on gender, age, race, education, party identification and political interest. The total N is 800, large enough to detect effects of r = .10 with 80 percent power (sample size required for r = .10, alpha = .05, and beta = .20, is N = 783). This study was approved by the University of Nebraska-Lincoln Institutional Review Board (Project ID: 52013).

Given that no validated batteries of the impact of politics on health exist, our survey instrument had to be created from scratch, and the sample described above is the first to respond to our survey items. Our survey instrument, however, is anchored in pre-existing and widely used diagnostic batteries. Specifically, as a starting point we employed the diagnostic instruments used by Alcoholics Anonymous (AA) and Gamblers Anonymous (GA), and adapted and modified the questions to probe for the impact of politics. For example, a question common to both the AA and GA surveys is, “Does gambling [your drinking] ever cause you to have difficulty sleeping.” In our survey we have a similar item: “I have lost sleep because of politics,” with response categories ranging from “strongly agree” to “strongly disagree.” We drafted 32 such items designed to probe the impact of politics in four broad categories: physical health, emotional health, regretted behavior, and social/lifestyle costs.

It is possible people will misattribute the causes of emotional or physical distress, blaming politics for unhappiness that actually stems from a different source. Our goal, however, is not to ascertain the degree to which politics is objectively causing particular problems but rather the degree to which people perceive politics as the source of those problems. This is consistent with longstanding patterns in research on gambling and alcoholism which typically investigate self-report attributions [17] because effective strategies to alleviate a person’s problems are intimately connected to that person’s perceptions of the source of those problems.

Our survey also included variables designed to capture a wide range of individual traits and characteristics such as socio-demographics (race, income, age, gender, employment status, and frequency of church attendance), personality (the Ten-Item Big-5 Personality Inventory and an abbreviated version of Altemeyer’s dogmatism battery), political attitudes (partisanship; ideology; disapproval/approval of Donald Trump; preferences on social and economic issues; attitudes toward those who hold opposing political beliefs), political knowledge (score on a five-item test of political knowledge), political interest (a four-point, single item), and political activity (frequency of political discussions, self-reported voting frequency, and five participatory behaviors–attending a political meeting, working in a political campaign, making a campaign contribution, holding any form of public office, and contacting a public official). Table 1 reports measurement, coding information, and descriptive statistics for all variables used in our analysis.

**Table 1:** Variables and measures.

Outcome Variables	Measurement and Coding	Mean (SD)
**Physical Health**	6-item additive index; range 6–30	12.96 (5.3)
**Emotional Health**	8-item additive index; range 8–39	19.28 (6.58)
**Regretted Behavior**	10-item additive index; range 10–46	21.69 (7.49)
**Lifestyle/Social Costs**	8-item additive index; range 8–40	15.24 (5.92)
**Comprehensive Battery**	32-item factor score; range -1.5–3.60	00.00 (.97)
**Short Battery**	10-item additive index (see text); range 10–49	24.50 (8.7)
**Outcome Co-Variates**		
***Socio-Demographic***		
**White**	1 = White 0 = other	.74 (.45)
**Black**	1 = Black 0 = other	.09 (.28)
**Hispanic**	1 = Hispanic 0 = other	.09 (.28)
**Asian**	1 = Asian 0 = other	.02 (.14)
**Income**	1–16 family income index where 1 = < $10K 16 = >$500K; range 1–16	5.61(3.4)
**Age**	Age in years; range 18–90	47.64 (17.15)
**Education**	6 item index where 1 = No HS 6 = post grad; range 1–6	3.27 (1.45)
**Unemployed**	1 = laid off or unemployed 0 = employed/retired/student	.10 (.29
**Male**	1 = Male 0 = other	.49 (.50)
**Church Attendance**	6-item scale, 1 = never 6 = more than once a week; range 1–6	2.79 (1.73)
***Personality***		
**Agreeableness**	TIPI 2 item index; range 2–10	7.29 (1.47)
**Emotional Stability**	TIPI 2 item index; range 2–10	7.12 (1.68)
**Dogmatism**	Altemeyer 2 item Dogmatism battery; range 2–10	5.23 (1.40)
***Political Attitudes***		
**Partisanship**	7-point scale, 1 = strong Dem 7 = Strong Rep; range 1–7	3.66 (2.12)
**Ideology**	3-point scale, 1 = lib, 2 = mod, 3 = con; range 1–3	2.03 (.77)
**Social Issues**	4-item additive social issues (death pen, gun control, gay marriage, immigration); range 4–20	12.07 (4.12)
**Economic Issues**	4 item additive econ issue (healthcare, small govt., welfare spending, lower taxes): range 4–20	11.4 (3.79)
**Trump Disapproval**	5-point scale, 1 = strongly approve 5 = strongly disapprove; range 1–5	3.32 (1.59)
**Political opposites**	3-item index of attitudes on political opposites (more informed, more close minded, less truthful); range 3–15	10.53 (2.71)
***Political Knowledge/ Interest***		
**Political Knowledge**	score on 5 question political knowledge quiz (who is current leader of Russia, who is current vice president, what fraction of voted need to override a veto, and which party has majority in Congress); range 0–5	3.14 (1.72)
**Political Interest**	4-item scale, 1 = low 4 = high; range 1–4	3.20 (.94)
***Political Engagement and Participation***		
**Voting Frequency**	5-item scale, 1 = never voted 5 = vote in every election; range1-5	4.03 (1.30)
**Political Participation**	No of activities participated in (pol mtg; camp. work, given money, held office, contacted official; range 0–5	1.57 (1.55)
**Discuss Politics**	4-item scale, 1 = never 4 = very often; range 1–4	2.72 (.86)

Total N = 800

## Results

Psychometric analysis indicated high internal consistency for the items within each of our four categories of physical health, emotional health, regretted behavior, and social/lifestyle costs (Cronbach’s alphas are reported in Table 2), and subjecting the items to factor analysis confirmed a single underlying dimension for each category. Given this, we used the items to create four separate additive indexes, one for each category. As all four batteries were strongly correlated with each other (r = .75 or higher, p. < .01) and all 32 of the individual items were positively correlated with each other (range approximately r = .20 to r = .75, p < .05), we suspected that our 32-items were picking up a single underlying dimension of the overall impact of politics.

**Table 2:** The costs of politics.

Item	Percent Agreeing	Item-Index Correlation
**Physical Health**		
Politics has caused me to be stressed.	38	0.79
I have become depressed when a preferred candidate lost.	26.4	0.77
Politics has caused me to be fatigued.	21.4	0.8
I have lost sleep because of politics.	18.3	0.82
Politics has adversely affected my physical health, even if only a little.	11.5	0.77
Politics has caused me to be suicidal.	4.1	0.55
*Cronbach's Alpha for all health costs items =* .*85*		
**Emotional Health**		
Exposure to media outlets promoting views contrary to mine can drive me crazy.	31.8	0.68
I have lost my temper as a result of politics.	29.3	0.73
Politics has led me to hate some people.	26.5	0.73
Politics has caused me to think seriously about moving.	23.3	0.64
On occasion, I have regretted comments I made during a political discussion.	18	0.6
I have secretly wished bad things on those who disagree with me politically.	15.3	0.71
I sometimes feel guilty about the way I feel toward those who disagree with me.	14.1	0.62
I have become annoyed when others are critical of my political views.	11	0.71
*Cronbach's Alpha for all emotional costs items =* .*83*		
**Regretted Behavior**		
I spend more time thinking about politics than I would like.	25.6	0.73
I care too much about who wins and loses in politics.	22.1	0.63
My life would be better if I didn't focus so much on politics.	16.8	0.65
At times, I wish I would have restrained myself more in political conversations.	16.4	0.67
I have posted or written things on-line that I later wished I hadn't.	15	0.67
I have vowed to spend less time on politics but failed to follow through.	13.1	0.72
I spend more time on political websites than I should.	12.4	0.71
Politics has sometimes caused me to exercise bad judgment.	8.9	0.68
My interest in politics has delayed me from completing an assignment, task, or job.	8.5	0.71
After a major election or political event, there is sometimes a void in my life.	11	0.65
*Cronbach's Alpha for all regretted behavior items =* .*87*		
**Social and Lifestyle Health**		
Differences in political views have damaged a friendship I valued.	20.3	0.70
Differences in political views have created problems for me in my extended family.	16.9	0.70
On occasion, politics has made my home life less pleasant.	16.6	0.71
Differences in political views have created problems for me in my immediate family.	14.6	0.76
Differences in political views have created problems for me at work.	8	0.69
I have lost time from work or school because of politics.	6	0.71
My political views have created financial problems for me.	4.8	0.72
My political views have created legal problems for me.	4.3	0.68
*Cronbach's Alpha for all social and lifestyle costs items =* .*86*		

Percent Agreeing = percent “agree” plus “strongly agree” for each item

Item/Index Correlation = correlation (Pearson’s r) between individual item and summative index. All reported coefficients p < .05.

Factor analysis provided mixed results for this expectation—different analytical approaches consistently yield a single, dominant dimension but there were also occasional hints of other dimensions. For example, an unrestricted maximum likelihood factor analysis with oblimin rotation resulted in two factors with eigenvalues greater than 1.0, but all 32-items loaded on the first factor at .49 or higher and there was no real theoretical coherence to the eight items that loaded at .30 or higher on the second factor. Accordingly, in the analysis reported below we used our four original indexes as the primary measures of health impact but we also included the overall measure containing all 32 items. Finally, recognizing that space in surveys is nearly always at a premium, we also broke out the 10 items with the highest percent agreeing (and thus with comparatively high variance) to create an abbreviated battery that could serve as an efficient instrument for measuring the health impacts of politics (Cronbach’s alpha for these ten items = .88, factor analysis yields a single dimension accounting for 44 percent of variance; see Table 3 for a complete list of the short battery items).

**Table 3:** 10-Item costs of politics battery.

Item	Percent Agreeing	Item-Index Correlation
Politics has caused me to be stressed.	38	0.78
I have become depressed when a preferred candidate lost.	26.4	0.73
Politics has caused me to be fatigued.	21.4	0.72
Exposure to media outlets promoting views contrary to mine can drive me crazy.	31.8	0.66
I have lost my temper as a result of politics.	29.3	0.73
Politics has led me to hate some people.	26.5	0.69
Politics has caused me to think seriously about moving.	23.3	0.62
I spend more time thinking about politics than I would like.	25.6	0.70
I care too much about who wins and loses in politics.	22.1	0.58
Differences in political views have damaged a friendship I valued	20.3	0.63
*Chronbach’s alpha =* .*88*		

Percent Agreeing = percent “agree” plus “strongly agree” for each item

Item/Index Correlation = correlation (Pearson’s r) between individual item and summative index. All reported coefficients p < .05.

These are the ten items with the highest “percent agreeing” from Table 2.

Table 2 reports the percent of respondents agreeing/strongly agreeing with each item, and these results consistently indicate politics exacts a toll on well-being. In terms of physical health, nearly 40% of respondents report experiencing stress as a result of politics, while roughly a fifth or more report losing sleep, being fatigued, or suffering depression because of politics. More than 10% report non-specific physical health issues related to politics and four percent even indicate they have considered suicide as a result of politics. Approximately 10 to 30% of the population also believed that politics took an emotional toll on them by triggering anger, frustration, hate, guilt, or leading to comments they later regretted.

A fairly large fraction of our respondents—roughly 10 to 25%—reported thinking, caring, and focusing on politics more than they want, saying and writing things they later regret, making bad decisions, ignoring other priorities, and feeling empty at the end of major political events. This suggests that a non-trivial number of people recognize certain forms of political involvement will exact significant personal costs, but have a hard time over-riding their inclination to engage and we refer to this as “regretted behavior.” Finally, about a fifth of our sample reported that politics had damaged friendships and created problems with family, friends, and in the home. Approximately five percent believed politics led to financial or legal problems or caused them to miss time at work or school.

Table 4 reports correlational analyses (Pearson’s r) between our primary “potential costs” measures and the individual-level traits and characteristics described above. These results indicate a number of patterns. First, across rows the coefficients display stable directions and comparable effect sizes. Seventeen of the 24 variables listed in the first column of Table 4 have consistent directional relationships with all six health measures. Eighteen variables display statistically significant correlations with at least two of the health measures and 15 of those show directional stability across the row. In short, regardless of the health measure examined, correlates seem to be highly comparable which again suggests that these measures are tapping into a single underlying dimension.

**Table 4:** Bivariate correlates of politically-related health measures.

	Physical	Emotion	Regret	Social/Lifestyle	10-item	32-Item
**Socio-Demographics**						
**White**	.01	.03	-.01	-.06	.05	-.02
**Black**	-.07	**-.08**	-.04	-.01	**-.10**	-.05
**Hispanic**	.01	-.02	.00	.04	-.02	.01
**Asian**	-.00	.00	.00	-.00	-.00	.00
**Income**	.05	.07	.03	-.01	**.12**	.03
**Age**	**-.18**	**-.16**	**-.12**	**-.21**	**-.12**	**-.20**
**Education**	**.09**	**.09**	.04	.01	**.11**	.06
**Unemployed**	.06	.06	.06	**.09**	.06	**08**
**Male**	.04	**.10**	**.14**	**.12**	**.08**	**.12**
**Personality**						
**Agreeableness**	**-.15**	**-.23**	**-.25**	**-.25**	**-.17**	**-.25**
**Emotional Stability**	**-.24**	**-.20**	**-.22**	**-.20**	**-.20**	**-.24**
**Dogmatism**	**.10**	**.20**	**.21**	**.15**	**.14**	**.19**
**Political Attitudes**						
**Partisanship**	**-.22**	**-.11**	**-.10**	**-.10**	**-.17**	**-.15**
**Ideology**	**-.29**	**-.18**	**-.13**	**-.16**	**-.25**	**-.21**
**Social Issues**	**-.34**	**-.19**	**-.14**	**-.17**	**-.26**	**-.23**
**Economic Issues**	**-.25**	**-.12**	**-.08**	**-.12**	**-.17**	**-.16**
**Trump Disapproval**	**.30**	**.20**	**.13**	**.15**	**.25**	**.21**
**Political opposites**	**.10**	**.20**	.06	.01	**.22**	**.09**
**Political Knowledge/Interest**						
**Political Knowledge**	.01	**.12**	-.02	**-.10**	**.16**	-.01
**Political Interest**	**.17**	**.21**	**.19**	**.07**	**.30**	**.17**
**Discuss Politics**	**.29**	**.29**	**.27**	**.21**	**.37**	**.28**
**Voting Frequency**	.06	**.10**	.07	-.01	**.15**	.05
**Political Participation**	**.28**	**.24**	**.25**	**.23**	**.32**	**.27**
**Church Attendance**	-.05	-.06	.06	.04	**-.07**	.00

Cell entries are simple correlation coefficients (Pearson’s r), bolded cell entries p < .05, N = 800

The second key result from Table 4 is the consistent pattern in the correlates of the health measures. Three socio-demographic traits co-vary with health impacts. Being unemployed is consistently associated with negative impacts and the negative coefficient for age and the positive coefficient for gender (male) suggests younger people and males are more likely than older people and females to suffer the negative costs of politics. In addition, two of the “Big Five” personality dimensions correlate with the costs inflicted by politics (the other three Big Five traits had no relationship and were excluded from the table). People scoring high on agreeableness are less likely to report maladies resulting from politics as are people scoring high in emotional stability, with the latter notable for its relatively large effect size. Being more dogmatic, on the other hand, is associated with a greater perceived toll of politics on well-being.

The five political attitudes included in our analysis show a strong and consistent relationship with all the health measures. Specifically, those on the political right report fewer negative impacts than those on the political left. Democrats, self-identified liberals, those who are socially and economically liberal, and people who disapprove of President Donald Trump are, across the board, more likely to report negative health impacts from politics. Beyond left-right orientation, other political variables are associated with negative health impact. People who frequently discuss politics and who report being more involved in politics are also more likely to score higher on all indexes. These relationships are not only statistically significant but demonstrate the largest effect sizes in our analysis (across all six health measures reported in Table 4, the mean coefficient for frequency of political discussion is r = .28, and the mean coefficient for political participation across all six health measures is r = .26). Similar effects are evident for those who claim to have high levels of interest in politics. People who hold harsher views of their political opposites also tend to report that politics has negative impacts on their lives. Interestingly political knowledge is one of the few variables showing an inconsistent relationship (direction and significance) with the political-related health measures. Note, however, that political knowledge measures based on the sort of “pop quiz” items used here have been criticized for failing to capture the general concept of practical, politically-relevant information held by citizens [18–19]; thus, the inconsistency we see here may reflect some of those underlying measurement concerns.

The takeaways from the simple bivariate analyses presented in Table 4 are fairly clear. The negative costs of politics tend to be associated with particular socio-demographic traits (age, employment, gender), political attitudes and orientations (a higher dislike of the political “other,” more liberal political orientations), as well as greater engagement with and attention to the political world (frequency of discussing politics, political participation, political interest). Do those relationships hold in more rigorous multivariate analyses?

Table 5 presents a series of regression models that seek to answer this question. The dependent variables in these models are the 10-item and 32-item indexes and the predictors are the same variables reported in Table 4 with one exception. The variables on political attitudes (partisanship, ideology, social conservatism, economic conservatism, Trump disapproval) were unsurprisingly strongly correlated with each other (r ≈ .60-.75). Regression diagnostics suggested there was no overwhelming problem with multicollinearity in the models themselves (variance inflation factors ranged from approximately 1.5 to 3.0), but a factor analysis (maximum likelihood with an oblimin rotation) of the political attitude variables suggested a single underlying dimension that picked up 65 percent of the variance. Accordingly, we re-ran the models dropping the individual variables and using the factor scores to represent a general liberal-conservative dimension (high numbers = more conservative). In the table we report standardized betas rather than unstandardized coefficients to help facilitate comparisons across rows and within columns.

**Table 5:** Multi-variate analysis of politically-related health measures.

Variable	32-item	10-item	32-item	10-item
**Black**	-.06	-.07	-.06	-.07
**Hispanic**	-.23	-.01	-.02	-.01
**Asian**	.00	.00	.00	.01
**Income**	.01	.05	.02	.06
**Age**	**-.09**	**-.09**	**-.10**	**-.09**
**Education**	-.04	-.05	-.04	-.05
**Unemployed**	**.11**	**.11**	**.11**	**.10**
**Male**	.02	-.01	.02	-.02
**Agreeableness**	**-.12**	**-.07**	**-.12**	**-.07**
**Emotional Stability**	**-.18**	**-.18**	**-.18**	**-.18**
**Dogmatism**	**.11**	**.09**	**.11**	**.09**
**Partisanship**	.01	.01	__	__
**Ideology**	-.06	-.05	__	__
**Social conservatism**	**-.12**	**-.11**	__	__
**Economic conservatism**	.07	.06	__	__
**Trump Disapproval**	**.11**	**.15**	__	___
**Combined Political Attitudes Measure**	**__**	**__**	**-.19**	**-.22**
**Political Opposites**	**.08**	**.14**	**.08**	**.15**
**Political knowledge**	-.09	-.00	-.07	.02
**Political Interest**	.05	**.12**	.04	**.10**
**Voting behavior**	-.02	-.00	-.01	.00
**Discuss Politics**	**.21**	**.20**	**.21**	**.20**
**Political Participation**	**.22**	.**19**	**.22**	**.19**
**Church Attendance**	.05	.00	.05	.00
**N**	645	645	645	645
**F**	**14.57**	**17.06**	**17.13**	**19.99**
**Adj R-2**	.33	.36	.32	.36

Cell entries are standardized correlation coefficients (i.e. standardized beta) from ordinary least squares regression model, bolded entries p < .05

The results of these models largely confirm the inferences from Table 4. In terms of socio-demographics, age and unemployment remain significant predictors, though gender does not. Personality traits tell the same story as in Table 4, as do attitudes towards political opposites. Social conservatism and Trump disapproval are significant and in the same direction reported above. Partisanship, ideology and economic conservatism are not significant predictors when the other variables are included. In models using the factor score of liberal-conservative attitudes described above, the measure is significant and the effect size suggests a one standard deviation in political orientations is associated with a fifth to a quarter of a standard deviation shift in the dependent variable. Political participation and frequency of discussing politics are consistent and significant predictors and, again, have some of the largest effect sizes.

The basic summary of the results presented in Table 5 is that they largely confirm the bivariate relationships reported in Table 4. Coding decisions on variables such as education and church attendance (i.e. the decision to treat them as continuous variables as reported in Table 5, or recode them into categorical variables and re-run the models) do not change any of the substantive inferences. As of early 2017, the patterns in our data clearly and consistently suggest that people who are more likely to report negative health-related impacts from politics are younger, unemployed, more dogmatic, more liberal, have relatively low opinions of their political opposites, discuss politics frequently, and participate at high rates. Two personality traits—agreeableness and emotional stability—are associated with experiencing fewer of these negative outcomes.

### Limitations

Before discussing the implications of our findings it is important to note some important limitations on our measures and data. To the best of our knowledge, this is the first attempt to comprehensively capture the social, psychological, and physical health costs and that necessarily meant drafting and fielding a large number of items. In any survey, item wording and order have the potential to distort results and these concerns certainly apply here. In particular, by following the format adopted in surveys of people who may be experiencing problems with alcohol usage or gambling (as well as the format used in the negative emotions battery of Gallup’s “Global Emotions Report”) our results may be altered by acquiescence bias. Since items in all these batteries are structured such that agreement indicates problems with gambling or alcohol or politics, a tendency on the part of respondents to agree with survey items would inflate estimations of the seriousness of these problems. The fact that we asked a large number of items (32) in quick succession could exacerbate this acquiescence bias. On the other hand, it has been suggested to us that the desire of many people to convey the impression that they can handle problems could easily lead to an underestimation of the degree to which politics creates difficulties in their lives. Be this as it may, readers should recognize that the results we offer could well be affected by acquiescence and other biases.

A second potential issue is our sample. The YouGov sample was both large (N = 800) and structured to be representative of American adults but is drawn from a pre-recruited, on-line panel and concerns about such sampling procedures rightly persist. Though research suggests internet panels produce results comparable to more traditional survey sampling methods such as random digit dialing [20–21], tradeoffs on representativeness and response quality are inevitable [22]. In the face of the widely-acknowledged problems of conducting traditional phone surveys in the cell-phone era, the internet panel option seems reasonable. Nonetheless, though we can defend our results as reasonably representative of the population of American adults, until they are replicated with other samples (and perhaps other sampling methods) they should be treated with appropriate caution.

Finally, we posed the items introduced here at only one point in time (March of 2017), meaning that any conclusions drawn concerning the correlates of people who believe they have suffered as a result of politics are not generalizable. We present the correlational results (Tables 4–5) largely to illustrate the approaches that we believe could be valuable once assessment of the perceived costs of politics is made at other times and in other national contexts. Our primary goal in this article is to introduce and to demonstrate the utility of these items.

## Discussion

Political interactions and experiences impact institutions and elites but also ordinary individuals. The costs of exposure to politics extend to social, mental, emotional, and physical health and the results presented here indicate that these costs are fairly widespread. Our sample was designed to be representative of the approximately 248 million adults living in the United States. Important caveats always attend when extrapolating from samples to populations but if our findings are even remotely externally valid then tens of millions of American adults perceive politics as exacting significant social, psychological and even physical health costs. Rough estimates based on Table 2 would be that approximately 94 million people believe they have been stressed by politics, 44 million believe they have lost sleep, 28.5 million that their physical health has been adversely affected, and 11 million that politics led them to consider suicide, though particular caution surrounds behaviors that are less common. For example, the 4.1 percent who report suicidal thoughts as a result of politics are less than 40 respondents in our sample, so even small amounts of sampling error could significantly change proportional estimates. Still, even the most conservative interpretation of these numbers suggests that large numbers of Americans are convinced that politics is exacting significant social, psychological and even physical costs on their well-being.

To try and put our results in comparative perspective we used the National Longitudinal Study of Adolescent to Adult Health [23] as a point of comparison. Though administered to young adults rather than a representative sample of all ages, this is a reliable and respected data source that asks a large number of roughly parallel questions on the effects of alcohol consumption. Approximately 11% of the Add Health respondents reported that alcohol use had created problems with family, friends, or people at work, which is slightly less than the numbers for parallel political items (see Table 2). In other areas, 15.4% of the Add Health sample reported that alcohol use interfered with responsibilities at work or school. In our sample, 6% reported losing time from work or school because of politics and 8.5% said politics delayed them from completing an assignment, task, or job. Further, 9.1% of the Add-Health sample reported legal problems because of drinking, presumably mainly as a result of DUIs and similar offenses. In our sample 4.3% said politics had created legal problems for them.

Our results also provide insight into the populations most likely to believe politics is adversely affecting them at the time the survey went to the field. They are younger and unemployed; more disagreeable (more critical and quarrelsome), and less emotionally stable (more anxious and easily upset). They tend to be politically liberal, strongly disapprove of President Donald Trump, and have low opinions of their political opposites (they see them as uninformed, closed-minded, and untruthful). They also tend to discuss politics frequently and to be actively involved in a range of political activities.

It may be that the costs of politics were unusually acute when this survey was administered, just two months after the inauguration of an extremely polarizing president. The fact that left-leaning people who disapproved of Trump were among the most consistent in reporting negative health costs raises the possibility that our sample is capturing a moment in time when costs for that group were particularly high. On the other hand, the presidencies of George W. Bush and Barack Obama were so deeply divisive [24] that each of the last three presidents was labeled, with empirical support, the most polarizing ever. Our expectation is that, while the ideological leanings of those most adversely affected by politics will change from administration to administration, the tendency of those who are most politically involved and interested to be most adversely affected will persist and this brings us to our final point.

Determining the extent to which the costs of politics vary across time and contexts is an important mission—one that we believe will be facilitated by our efforts to construct and validate appropriate survey batteries. We believe the batteries offered here will make it possible to take regular soundings of the perceived costs of politics, thereby building a knowledge base regarding the conditions under which politics is believed to be the most (and least) damaging to personal well-being. The 10-item battery in particular captures a wide range of costs and is concise enough to be included in most survey instruments.

Democratic governance brings undeniable benefits to its citizens and in some cases political involvement is undoubtedly beneficial to personal well-being [25]. At the same time, for many people election campaigns and the contentious discussion of issues and candidates that surrounds them bring undeniable costs. Only by identifying, measuring, and analyzing the personal costs of open, democratic politics will it be possible to ameliorate them.
